# YOLO-Extreme: Obstacle Detection for Visually Impaired Navigation Under Foggy Weather

**DOI:** 10.3390/s25144338

**Published:** 2025-07-11

**Authors:** Wei Wang, Bin Jing, Xiaoru Yu, Wei Zhang, Shengyu Wang, Ziqi Tang, Liping Yang

**Affiliations:** 1 College of Computer Science and Technology, Changchun University, No. 6543, Satellite Road, Changchun 130022, China; 2 College of Construction Engineering, Jilin University, Changchun 130026, China

**Keywords:** YOLO-Extreme, foggy environments, Dual-Branch Bottleneck Block, Multi-Dimensional Collaborative Attention Module, Channel-Selective Fusion Block, RTTS foggy dataset

## Abstract

Visually impaired individuals face significant challenges in navigating safely and independently, particularly under adverse weather conditions such as fog. To address this issue, we propose YOLO-Extreme, an enhanced object detection framework based on YOLOv12, specifically designed for robust navigation assistance in foggy environments. The proposed architecture incorporates three novel modules: the Dual-Branch Bottleneck Block (DBB) for capturing both local spatial and global semantic features, the Multi-Dimensional Collaborative Attention Module (MCAM) for joint spatial-channel attention modeling to enhance salient obstacle features and reduce background interference in foggy conditions, and the Channel-Selective Fusion Block (CSFB) for robust multi-scale feature integration. Comprehensive experiments conducted on the Real-world Task-driven Traffic Scene (RTTS) foggy dataset demonstrate that YOLO-Extreme achieves state-of-the-art detection accuracy and maintains high inference speed, outperforming existing dehazing-and-detect and mainstream object detection methods. To further verify the generalization capability of the proposed framework, we also performed cross-dataset experiments on the Foggy Cityscapes dataset, where YOLO-Extreme consistently demonstrated superior detection performance across diverse foggy urban scenes. The proposed framework significantly improves the reliability and safety of assistive navigation for visually impaired individuals under challenging weather conditions, offering practical value for real-world deployment.

## 1. Introduction

Navigating safely and independently is a persistent challenge for visually impaired individuals, particularly when they are exposed to complex and unfamiliar environments [1,2]. While assistive technologies based on computer vision have significantly advanced obstacle detection under typical conditions [3,4], foggy weather introduces additional hazards that seriously compromise both perception and safety [5]. In these adverse environments, obstacles can become visually indistinct, backgrounds turn ambiguous, and even commonly reliable cues may disappear altogether. These factors not only diminish the effectiveness of conventional detection systems but also dramatically increase the risk of accidents and reduce the confidence of visually impaired people to travel alone [6,7]. Thus, it becomes crucial to develop robust obstacle detection methods specifically designed to address the unique challenges posed by foggy weather, with the ultimate goal of improving mobility and quality of life for the visually impaired community [8].

Visually impaired individuals face even greater challenges in adverse weather conditions, where traditional mobility aids such as guide dogs [9] and white canes [10] become increasingly inadequate. Guide dogs, while providing valuable support, are unable to detect environmental changes or obstacles that are visually obscured by fog, and their effectiveness is further limited by high training costs and maintenance requirements [11]. White canes, which are already restricted to detecting obstacles within a short range, are particularly ineffective in situations where obstacles are visually hidden or when tactile feedback is compromised by slippery or obstructed surfaces. Recognizing these limitations, some researchers have begun to explore technological solutions aimed at addressing the unique difficulties posed by foggy weather for the visually impaired. For example, Chu et al. [12] proposed D-YOLO, which employs a dual-branch network and feature-level adaptation to improve object detection in adverse weather conditions. Despite these efforts, most existing systems still fall short in providing robust and reliable obstacle detection under adverse foggy conditions, highlighting the urgent need for improved methods that can enhance both the safety and independence of visually impaired individuals during inclement weather [13].

Among the various approaches to object detection, one-stage and two-stage detectors have become the two dominant paradigms. One-stage object detectors, such as those in the YOLO [14] and SSD [15] families, are designed to predict object categories and bounding boxes in a single forward pass through the network. This architectural simplicity yields high inference speed, making one-stage detectors particularly well-suited for real-time applications and resource-constrained devices, such as wearable systems for visually impaired individuals. In contrast, two-stage object detectors, exemplified by the R-CNN series [16,17], decompose the detection process into an initial stage that generates region proposals and a subsequent stage that performs fine-grained classification and localization. Although two-stage detectors often achieve higher accuracy, their increased computational complexity can pose challenges for real-time deployment. In the context of assistive navigation under extreme weather conditions, the choice between these two frameworks becomes even more critical, as the detection system must balance robustness, accuracy, and efficiency to meet the demands of practical, real-world scenarios [18].

**Figure 1:**
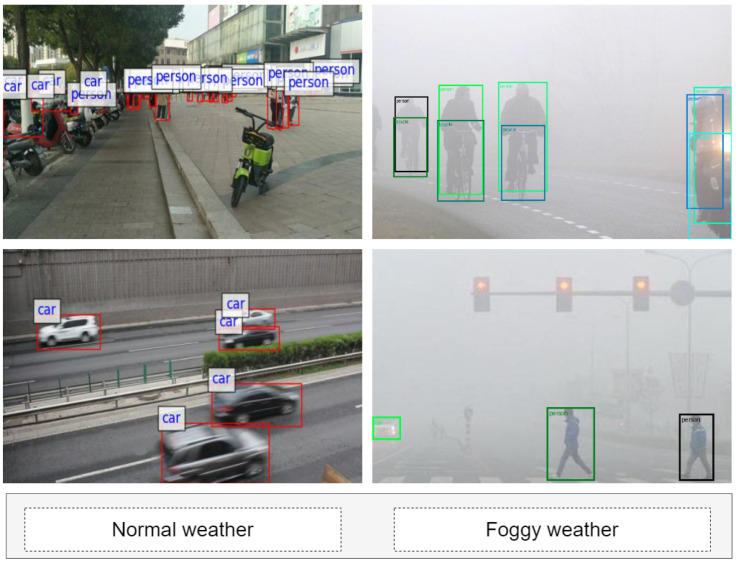
Visualization of object detection in different weather conditions.

As illustrated in Figure 1, obstacle detection systems for visually impaired navigation encounter a range of significant challenges when transitioning from normal to foggy weather conditions. Under clear weather, obstacles such as pedestrians, vehicles, and bicycles present distinct boundaries and salient features, allowing for reliable detection. However, in foggy scenes, object boundaries become blurred and essential visual cues are greatly weakened, making it increasingly difficult for detection algorithms to distinguish obstacles from the background and leading to a higher likelihood of missed detections. At the same time, adverse weather introduces substantial background noise, which often generates dynamic artifacts resembling real obstacles, thereby increasing the rate of false positives. The inherent complexity and dynamic variability of real-world navigation scenes, including moving vehicles, groups of pedestrians, temporary obstructions, and fluctuating lighting, further exacerbate the instability of detection systems under such challenging conditions. These phenomena, clearly observed in Figure 1, highlight the urgent need for advanced, weather-adaptive obstacle detection frameworks capable of maintaining robust and accurate performance across a wide spectrum of adverse environments [19].

**Figure 2:**
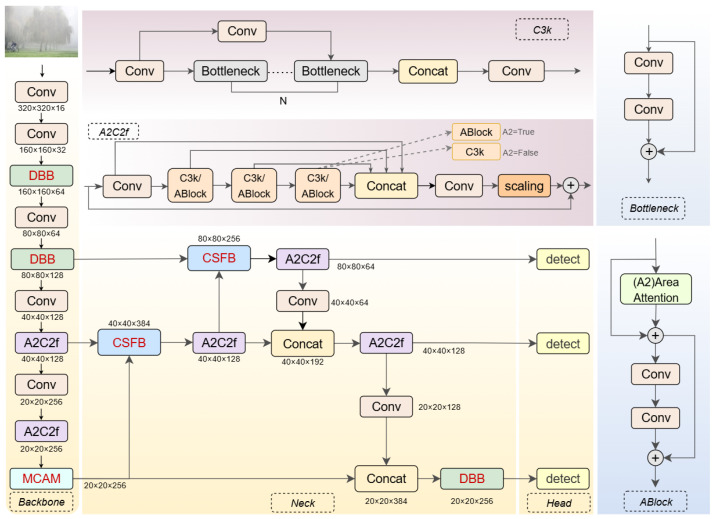
Architecture of the proposed method.

To address the complex challenges of obstacle detection for visually impaired navigation under foggy weather conditions, we propose YOLO-Extreme, an enhanced YOLOv12-based framework [20]. As illustrated in Figure 2, the proposed architecture incorporates three key modules: the Dual-Branch Bottleneck Block (DBB) [21], the Multi-Dimensional Collaborative Attention Module (MCAM) [22], and the Channel-Selective Fusion Block (CSFB) [23]. The DBB is integrated into both the backbone and neck of the network to simultaneously capture local spatial details and global semantic information, thereby improving the ability of the model to distinguish blurred or low-contrast obstacles caused by fog. The MCAM is placed at the end of the backbone and adaptively refines feature maps by aggregating attention across multiple spatial dimensions, which effectively suppresses background noise and emphasizes salient obstacle cues in cluttered scenes. The CSFB is embedded in the neck to dynamically recalibrate the channel-wise contributions of high- and low-resolution features, enabling robust and adaptive multi-scale feature fusion that is critical for stable detection in dynamic and complex environments.

To provide a concise overview of our contributions, we present them in five key aspects:

(1) We propose YOLO-Extreme, a novel object detection framework designed to address the challenges of foggy weather conditions in visually impaired navigation. Our method incorporates the Dual-Branch Bottleneck Block (DBB), the Multi-Dimensional Collaborative Attention Module (MCAM), and the Channel-Selective Fusion Block (CSFB) for enhanced robustness and accuracy.
(2) To effectively extract both local spatial and global semantic features, we integrate the Dual-Branch Bottleneck Block (DBB) into the backbone and neck, which significantly improves the discrimination of blurred or low-contrast obstacles caused by fog.
(3) To suppress background noise and highlight salient obstacle cues in cluttered environments, we introduce the Multi-Dimensional Collaborative Attention Module (MCAM), which adaptively aggregates spatial attention from multiple dimensions to enhance feature representation under foggy weather conditions.
(4) To enable robust and adaptive multi-scale feature fusion, the Channel-Selective Fusion Block (CSFB) is incorporated into the neck, dynamically recalibrating channel-wise contributions from high- and low-resolution features, and thereby stabilizing detection performance in complex and dynamic scenes.
(5) Experimental results on the publicly available RTTS dataset [24] and Foggy Cityscapes dataset demonstrate that YOLO-Extreme achieves superior performance in obstacle detection under foggy weather conditions, validating its potential as a practical navigational aid for visually impaired individuals.

## 2. Related Work

### 2.1. Advances in Object Detection

The development of object detection has seen a rapid evolution from early handcrafted features to advanced deep learning-based frameworks [25]. Deep convolutional neural networks now dominate this field, with algorithms typically divided into two primary streams: one-stage detectors and two-stage detectors. One-stage models, such as YOLO [26,27,28] and SSD [15], are designed for speed, integrating category prediction and bounding box regression into a single forward pass. This makes them especially attractive for applications requiring real-time feedback, such as wearable assistive devices. Two-stage models, including Faster R-CNN [16] and its derivatives [17], separate region proposal from classification and localization, often yielding improved precision at the cost of increased computational demands. More recent innovations include anchor-free detectors [29] and transformer-based frameworks [30,31,32], which further expand the capabilities of detection systems. However, nearly all of these advances are optimized on datasets collected under normal visibility, leaving their robustness in challenging weather largely unexplored.

### 2.2. Detection in Challenging Weather

Detection in challenging weather remains a critical bottleneck for the practical deployment of computer vision systems, especially in outdoor or autonomous scenarios [33]. Adverse conditions such as fog can severely impair image quality by reducing contrast, blurring object boundaries, and introducing unpredictable visual artifacts. To address these issues, existing research has explored three main directions: image enhancement pipelines that apply dehazing or deraining algorithms prior to detection [34]; integrated networks that jointly optimize both enhancement and detection tasks [35]; and domain adaptation strategies aimed at bridging the distribution gap between clear and weather-degraded data [36]. While these methods have led to incremental improvements, they each have limitations: enhancement-based approaches may remove subtle features critical for detection, joint networks often incur high computational costs, and domain adaptation may not fully generalize to real-world weather variability. Notably, few studies address assistive navigation needs under such conditions.

### 2.3. Similar Works

Several recent studies have explored object detection in adverse weather using improved YOLO-based architectures and public benchmark datasets [33,37,38,39,40]. Chu et al. [12] proposed D-YOLO, a dual-branch network that leverages feature adaptation and attention-based fusion to enhance detection robustness in foggy and hazy conditions. Gharatappeh et al. [41] introduced FogGuard, which utilizes a teacher–student perceptual loss along with synthetic fog data augmentation to boost the accuracy of YOLOv3 on foggy images, without incurring additional inference. Ding et al. [42] developed CF-YOLO, incorporating a cross-fusion feature aggregation module and a graded real-snow dataset (RSOD) to address object detection under real-world snowy conditions. Sun et al. [43] proposed FDW-YOLOv8, which integrates feature-enhancing attention and dark channel prior defogging to improve pedestrian detection robustness in foggy scenes. While these approaches have advanced the state of object detection under challenging environmental conditions, their main application scenarios are typically general outdoor perception or autonomous driving. In contrast, our work reframes the detection task from the perspective of assistive navigation for visually impaired individuals, aiming to address the unique requirements for safety and real-time reliability in extreme weather environments.

## 3. Methodology

### 3.1. Architecture of the Proposed Method

YOLO-Extreme is designed to address the unique challenges of obstacle detection for visually impaired navigation in foggy and low-contrast environments. Building upon the attention-centric YOLOv12 framework, YOLO-Extreme adopts a modular architecture comprising a feature extraction backbone, a multi-scale fusion neck, and a multi-branch detection head, as illustrated in Figure 2.

In the backbone, the conventional C3K2 modules employed in YOLOv12 for hierarchical feature aggregation are replaced with Dual-Branch Bottleneck Blocks (DBB). While C3K2 modules are effective in aggregating local features through convolutional operations, they are limited in their ability to simultaneously capture fine-grained spatial cues and long-range semantic dependencies—attributes critical for distinguishing blurred and low-contrast obstacles under foggy conditions.

The proposed DBB addresses these limitations by employing a dual-branch structure: one branch specializes in local detail extraction via depthwise convolutions, while the other focuses on global semantic enhancement through pointwise convolutions and channel interactions. This synergistic design enables the backbone to construct richer and more discriminative feature representations, thereby enhancing the model’s robustness and accuracy in degraded visibility environments.

At deeper stages of the backbone, the Multi-Dimensional Collaborative Attention Module (MCAM) is introduced. MCAM jointly models spatial and channel-wise attention, adaptively highlighting salient obstacle features and suppressing irrelevant background information. This attention-guided refinement further strengthens the network’s capability to cope with background clutter and adverse weather.

For multi-scale feature fusion, the original YOLOv12 employs upsampling and straightforward concatenation to integrate features from different stages. In YOLO-Extreme, these traditional fusion operations are systematically replaced with Channel-Selective Fusion Blocks (CSFB). Rather than relying on simple spatial upsampling and direct concatenation, CSFB dynamically recalibrates the channel-wise significance of aggregated features, allowing for more discriminative and efficient integration of multi-scale information. This approach not only preserves critical contextual and detailed cues but also mitigates feature redundancy and information loss—resulting in a more robust fusion process that is well-suited to the complexities of real-world environments.

The final fused features are processed by parallel detection heads, which predict bounding box coordinates and class probabilities at multiple spatial resolutions. This multi-scale prediction mechanism ensures reliable detection of obstacles of various sizes and supports comprehensive scene understanding, which is vital for safe and efficient navigation assistance.

By systematically replacing the original C3K2, upsampling, and concatenation structures with DBB and CSFB modules, and further integrating MCAM for attention-guided refinement, YOLO-Extreme achieves a balanced synergy between accuracy and computational efficiency. The explicit modular innovations enable the network to overcome the intrinsic limitations of conventional detectors under challenging visibility conditions, providing a robust and practical solution for assistive navigation in adverse weather.

### 3.2. Dual-Branch Bottleneck Block

**Figure 3:**
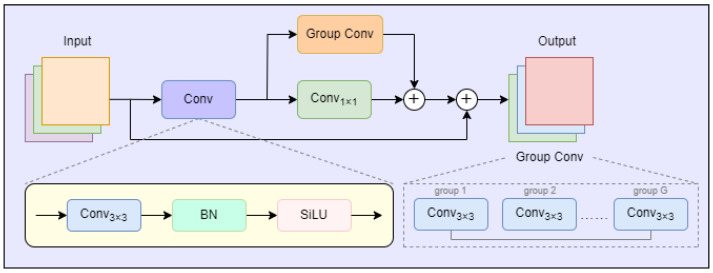
Architecture of the proposed Dual-Branch Bottleneck Block (DBB).

As illustrated in Figure 3, we propose that the Dual-Branch Bottleneck Block (DBB) [21] replace the C3k2 module of the YOLOv12 framework, which aims to address the challenge of feature degradation under foggy conditions. These conditions often cause object boundaries to become indistinct and textures to blur, which significantly reduces the reliability of obstacle detection for visually impaired individuals navigating real-world environments. The DBB adopts a parallel dual-branch architecture, consisting of a group convolution branch for local spatial feature extraction and a pointwise convolution branch for global semantic representation. The outputs of the two branches are fused via a residual connection, as shown in Figure 3. This design enables the network to robustly extract both local and global features in parallel, thereby improving detection accuracy and robustness under visibility-degraded conditions.

The DBB adopts a parallel dual-branch architecture to enhance feature learning. Specifically, the input feature map $X\in R^{C\times H\times W}$ is first processed by a shared transformation:

(1) $$X_{1}=\sigma \left(\mathrm{BN}\left({\mathrm{Conv}}_{3\times 3}\left(X\right)\right)\right)$$

where ${\mathrm{Conv}}_{3\times 3}\left(\cdot \right)$ denotes a 3×3 convolution, BN(·) is batch normalization, and σ(·) denotes the SiLU activation function. This operation produces an intermediate feature map $X_{1}$, which retains the spatial resolution of *X* and typically preserves the channel dimension ($C^{\prime }=C$).

The intermediate feature $X_{1}$ is then simultaneously passed through two independent branches:

Local branch: 3×3 group convolution, denoted as ${\mathrm{GroupConv}}_{3\times 3}\left(X_{1}\right)$, is used to capture fine-grained spatial features within channel groups. This operation is crucial for modeling local structure and edges that are easily degraded in foggy conditions, yielding output $X_{g}$.

Global branch: 1×1 pointwise convolution, denoted as ${\mathrm{Conv}}_{1\times 1}\left(X_{1}\right)$, is used to aggregate and redistribute information across all channels, capturing high-level semantic context and facilitating global feature interaction. The output of this branch is $X_{p}$.

The dual-branch processing is expressed as follows:

(2) $$X_{g}={\mathrm{GroupConv}}_{3\times 3}\left(X_{1}\right),\;X_{p}={\mathrm{Conv}}_{1\times 1}\left(X_{1}\right)$$

To fully exploit both local and global feature information, the outputs of the two branches are fused by element-wise addition and combined with the original input through a residual connection:

(3) $$Y=X_{g}+X_{p}+X$$

where $Y\in R^{C^{\prime }\times H\times W}$ is the final output of the DBB, integrating enhanced spatial and semantic features.

This design ensures that the network robustly extracts both local details and global semantics in parallel, effectively counteracting feature degradation caused by fog and low contrast. The explicit residual connection not only facilitates feature reuse and gradient flow but also stabilizes network training.

The DBB is systematically employed to replace all original bottleneck units in the C3K2 modules across both the backbone and the detection head, as shown in Figure 3. This modular replacement directly strengthens multi-scale feature extraction throughout the network, leading to improved obstacle detection performance under challenging weather conditions.

### 3.3. Multi-Dimensional Collaborative Attention Module

**Figure 4:**
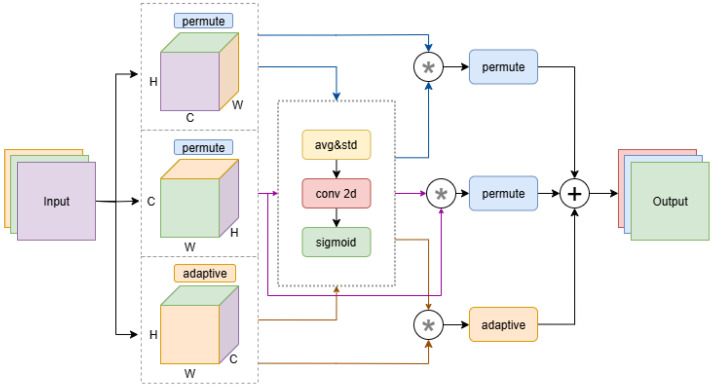
Multi-Dimensional Collaborative Attention Module.

As illustrated in Figure 4, to further enhance the feature extraction capability of the model in foggy weather and complex environments, we introduce a Multi-Dimensional Collaborative Attention Module (MCAM) [22] and integrate it at the end of the YOLOv12 backbone. The MCAM is designed to adaptively refine feature maps from multiple spatial perspectives, thereby improving the model’s robustness against blurred contours and background noise commonly encountered in real-world navigation scenarios for visually impaired individuals.

The MCAM explicitly processes features through a structured data flow as follows: The output feature map $X\in R^{C\times H\times W}$ from the backbone is provided as the input to MCAM.

Parallel branch processing: The input *X* is distributed to three parallel branches:

Height branch: *X* is permuted to (B,H,C,W), so that the height dimension acts as a “batch” for channel attention computation. This branch focuses on vertical structures, which is important for detecting elongated obstacles or those with partial occlusion.

Width branch: *X* is permuted to (B,W,H,C), capturing channel dependencies along the width dimension to enhance the identification of wide obstacles or objects with strong lateral features.

Channel branch: The original (B,C,H,W) layout is retained, and standard channel attention is applied to emphasize globally salient semantic information.

Attention generation and feature reweighting: Within each branch, the feature map is passed through a collaborative attention gate. Both the mean and standard deviation along the respective dimension are computed, concatenated, and processed with a 2D convolution and sigmoid activation to generate a dimension-specific attention map *A*:

(4) A=σ(Conv2D(Concat(AvgPool(X),StdPool(X))))

where the resulting attention map *A* is used to reweight the features within each branch, adaptively emphasizing informative elements and suppressing noise, the asterisk (*) denotes element-wise multiplication.

Feature alignment and fusion: After attention-based reweighting, the outputs from the height, width, and channel branches ($Y_{h}$, $Y_{w}$, $Y_{c}$) are permuted back to the original (B,C,H,W) shape. The three recalibrated features are then fused by element-wise addition and averaging:

(5) $$Y=\frac{1}{3}\left(Y_{h}+Y_{w}+Y_{c}\right)$$

where *Y* denotes the refined feature map output from MCAM.

Output: The final output *Y* is forwarded to the subsequent neck module for multi-scale feature fusion and downstream detection.

By explicitly structuring the data flow through parallel branches, attention generation, and feature fusion, MCAM enables the network to adaptively highlight critical obstacle features while suppressing environmental noise. This comprehensive design greatly improves detection robustness under diverse and degraded visual conditions, providing substantial benefit for reliable navigation assistance to visually impaired individuals in dynamic, real-world environments.

### 3.4. Channel-Selective Fusion Block

**Figure 5:**
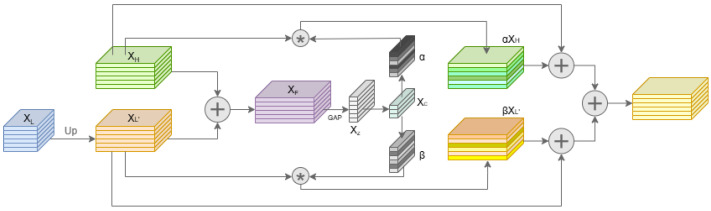
Channel-Selective Fusion Block (CSFB).

In real-world navigation scenarios, especially under foggy weather conditions, robust multi-scale feature fusion is essential for accurate obstacle detection. Traditional fusion methods based on simple upsampling and concatenation may amplify noise or dilute important features, limiting the reliability of detection under low-visibility conditions. To address this, we propose the Channel-Selective Fusion Block (CSFB) [23], as shown in Figure 5, which adaptively recalibrates the contributions of high- and low-resolution features through a dynamic channel attention mechanism.

Given a low-resolution feature map $X_{L}\in R^{C\times H_{L}\times W_{L}}$ and a high-resolution feature map $X_{H}\in R^{C\times H_{H}\times W_{H}}$, the CSFB first upsamples $X_{L}$ to match the spatial resolution of $X_{H}$, yielding $X_{L}^{\prime }=\mathrm{Up}\left(X_{L}\right)$. The upsampled low-resolution feature $X_{L}^{\prime }$ and the high-resolution feature $X_{H}$ are then fused by element-wise addition to obtain an initial combined feature:

(6) $$X_{F}=X_{H}+X_{L}^{\prime }$$

To extract global contextual information, global average pooling is applied to $X_{F}$, resulting in a channel descriptor $X_{z}=\mathrm{GAP}\left(X_{F}\right)$. This descriptor is subsequently compressed by a bottleneck fully connected layer and a nonlinear activation function, producing a compact embedding:

(7) $$X_{c}=\delta \left({\mathrm{FC}}_{1}\left(X_{z}\right)\right)$$

where δ(·) denotes a nonlinear activation such as ReLU.

To adaptively control the channel-wise contributions of the two input features, $X_{c}$ is expanded via two independent fully connected layers, generating channel attention weights α and β for the high- and low-resolution branches, respectively:

(8) $$\alpha =\sigma \left({\mathrm{FC}}_{a}\left(X_{c}\right)\right),\;\beta =\sigma \left({\mathrm{FC}}_{b}\left(X_{c}\right)\right)$$

where σ is the sigmoid activation and $\alpha ,\beta \in R^{C}$. These attention weights are used to recalibrate the original features through channel-wise multiplication:

(9) $${\hat{X}}_{H}=\alpha ⊙X_{H},\;{\hat{X}}_{L}^{\prime }=\beta ⊙X_{L}^{\prime }$$

where ⊙ denotes channel-wise multiplication.

Finally, the recalibrated features ${\hat{X}}_{H}$ and ${\hat{X}}_{L}^{\prime }$ are aggregated with the initial fused feature $X_{F}$ via a residual connection [44], producing the output of CSFB:

(10) $$Y=X_{F}+{\hat{X}}_{H}+{\hat{X}}_{L}^{\prime }$$

where $Y\in R^{C\times H_{H}\times W_{H}}$.

Throughout the process, each step ensures that spatial alignment, channel recalibration, and feature integration are coherently maintained. By dynamically emphasizing informative channels from both high- and low-resolution features while suppressing irrelevant noise, the CSFB significantly enhances the network’s robustness and reliability for obstacle detection in challenging, low-visibility environments.

## 4. Experiment

### 4.1. Datasets and Evaluation Metrics

All experiments in this study were conducted on the RTTS and Foggy Cityscapes datasets [24]. The RTTS dataset consists of 4322 real-world foggy traffic images captured in hazy weather conditions, with object annotations for five typical traffic categories: person, bicycle, car, bus, and motorbike. The annotations follow the COCO standard, facilitating fair comparison with existing methods for foggy scene understanding.

The Foggy Cityscapes dataset [45] contains 5000 synthetic foggy images generated from real Cityscapes urban scenes using physically based rendering. It provides fine-grained pixel-level annotations and bounding boxes for 19 semantic categories, including road, sidewalk, building, wall, fence, pole, traffic light, traffic sign, vegetation, terrain, sky, person, rider, car, truck, bus, train, motorcycle, and bicycle. In our work, we focused on the detection performance for five major traffic-related categories: person, bicycle, car, bus, and motorbike. This allows for a consistent and fair comparison with the RTTS dataset and ensures the evaluation addresses the most safety-critical targets in real-world navigation.

To quantitatively evaluate detection performance, we adopted several commonly used metrics. Specifically, precision (P) and recall (R) were computed as basic indicators of detection quality. In addition, average precision (AP) and mean average precision (mAP) are reported according to the COCO evaluation protocol, which averages AP over multiple intersection-over-union (IoU) thresholds from 0.5 to 0.95 with a step size of 0.05. The detailed formulations are as follows:

(11) $$P=\frac{TP}{TP+FP}$$

where TP denotes the number of true positives and FP is the number of false positives.

(12) $$R=\frac{TP}{TP+FN}$$

where FN is the number of false negatives.

(13) $$AP@0.5=\int_{0}^{1}P\left(R\right)\;dR$$

where the average precision (AP) at a given IoU threshold (for example, AP@0.5) is defined as the area under the precision–recall curve.

(14) $$mAP=\frac{1}{10}\sum_{i=0}^{9}AP{@}_{\left(0.5+i\times 0.05\right)}$$

where the mean average precision (mAP) is then calculated by averaging AP across multiple IoU thresholds.

These metrics comprehensively measure both the localization and classification performance of the object detection models, providing a reliable basis for comparison under challenging, real-world weather conditions.

### 4.2. Implementation Details

All experiments were conducted using the Ultralytics YOLO framework on a single NVIDIA RTX 3090 GPU. The proposed models were trained for 300 epochs using the SGD optimizer, with an initial learning rate of 0.01 and a batch size of 32. The input image size was set to 640 × 640 pixels. The RTTS dataset, formatted in COCO style, was used for both training and validation. During training, mixed precision (AMP) was disabled to ensure stable convergence. The confidence threshold for visualization and evaluation was set to 0.5 by default. All other settings followed the official Ultralytics YOLOv12 implementation unless otherwise specified.

### 4.3. Ablation Experiments

To quantitatively assess the contribution of each proposed module, we conducted ablation experiments on the RTTS dataset under foggy weather conditions. Table 1 summarizes the mAP and AP for each category, while Table 2 presents the FLOPs and parameter counts.

**Effect of Dual-Branch Bottleneck Block (DBB):** Introducing the DBB module increased the overall mAP from 48.2% to 49.5%. Notably, the AP for the person category improved from 50.7% to 52.0%, and for the car category from 58.1% to 59.6%. The parameter count and FLOPs only slightly increased, from 2.51 M to 2.55 M and from 5.8 G to 6.3 G, respectively. This result demonstrates that DBB can significantly enhance the representation of blurred or low-contrast obstacles in foggy environments while incurring minimal resource overhead.

**Effect of Multi-Dimensional Collaborative Attention Module (MCAM):** Integrating the MCAM module further elevated the mAP to 49.2%. The AP for the car category increased from 58.1% to 59.7%, and for the bus category from 44.5% to 44.9%. Parameters and FLOPs only marginally increased to 2.56 M and 6.3 G. This suggests that MCAM effectively suppresses fog-induced background noise and adaptively highlights salient obstacle features, improving detection robustness under low visibility.

**Effect of Channel-Selective Fusion Block (CSFB):** With the addition of the CSFB module, mAP increased to 49.7%. AP for the car category increased from 58.1% to 60.3%, and for the bus category from 44.5% to 46.7%. Parameter count and FLOPs increased to 3.44 M and 9.1 G. CSFB enhanced multi-scale contextual modeling and improved the discrimination of overlapping or blurred objects, demonstrating strong value for urban navigation in foggy conditions.

When all three modules were combined, YOLO-Extreme achieved a mAP of 50.1%, with AP for the car and person categories reaching 60.5% and 53.0%, respectively. The total parameter count and FLOPs reached 3.45 M and 9.1 G, which remain feasible for real-time deployment. These results confirm that each proposed module offers complementary benefits, enabling robust and accurate obstacle detection for visually impaired navigation in foggy weather.

### 4.4. Comparative Experiments

To further demonstrate the practical value of our approach, we conducted comprehensive comparative experiments on the RTTS dataset, benchmarking against state-of-the-art dehaze-and-detect algorithms (e.g., GridDehaze-YOLOv8, DCP-YOLOv8) and mainstream object detection models (e.g., YOLOv8, YOLOv12). As summarized in Table 3 and Table 4, we report inference speed (Speed, FPS), overall mean average precision (mAP), and per-category AP, providing a holistic view of both efficiency and robustness in real-time obstacle detection for assistive navigation.

In terms of detection accuracy, our method achieved the highest overall mAP (0.501) among all methods, surpassing the best-performing baseline YOLOv12 (0.482) and significantly outperforming dehaze-and-detect approaches such as GridDehaze-YOLOv8 (0.371). For categories crucial to urban navigation—person, bicycle, car, motorbike, and bus—our method yielded superior AP values (e.g., car: 0.605, person: 0.530). This indicates enhanced reliability for detecting diverse obstacles under foggy conditions.

To further verify the generalization capability of our method, we also conducted cross-dataset experiments on the Foggy Cityscapes dataset. As shown in Table 5, our approach achieved the highest overall mAP (0.239) compared to other state-of-the-art methods, outperforming both classical detectors and recent dehaze-based frameworks. Notably, our model yielded the best AP scores for all major categories of interest (person: 0.457, bicycle: 0.163, car: 0.245, motor: 0.155, bus: 0.371), demonstrating consistent robustness and effectiveness in different urban foggy environments. These results confirm the strong generalization ability of our method and its practical value for real-world assistive navigation applications.

Regarding inference efficiency, our model achieved a processing speed of 0.0043 s/image (232 FPS), far exceeding most dehaze-based algorithms (e.g., GridDehaze-YOLOv8 at 14.1 FPS) and maintaining a real-time processing level comparable to YOLOv12 (365 FPS). This ensures timely obstacle awareness, which is essential for practical deployment in mobile or wearable assistive devices.

Overall, our method achieves an excellent trade-off between detection accuracy and inference speed, demonstrating strong application potential in real-world foggy environments, where both robust perception and low-latency response are vital for the safety and mobility of visually impaired individuals.

## 5. Visualization

**Figure 6:**
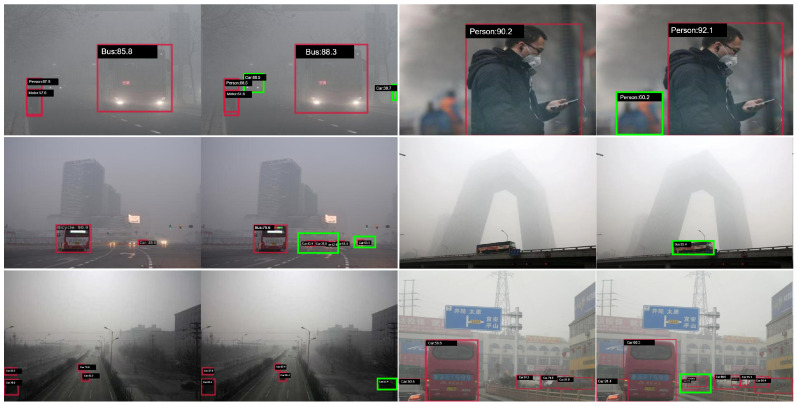
Visualization of detection results: YOLOv12 (**left**) vs. YOLO-Extreme (**right**). The green boxes indicate obstacles detected by our method but missed by the baseline model.

As illustrated in Figure 6, we provide a qualitative comparison of the detection results between the baseline YOLOv12 model (left) and our proposed method (right) under challenging foggy weather conditions. In each pair of images, the left side displays the results of YOLOv12, while the right side shows the results of our method. The red bounding boxes represent detections made by YOLOv12, and the green bounding boxes correspond to those identified by our method but missed by the baseline.

It can be observed that YOLOv12 struggles to accurately detect objects—such as vehicles, pedestrians, and bicycles—when visibility is significantly reduced due to dense fog. Many small or partially occluded obstacles are either not detected or are misclassified. In contrast, our method achieves more robust and precise detection results, successfully identifying critical obstacles with higher confidence, even in severe low-visibility scenarios. For instance, in the first row, the baseline fails to detect certain vehicles, whereas our approach accurately localizes both large and small objects. In the second and third rows, our method demonstrates clear advantages in detecting distant and partially obscured objects, which are often overlooked by the baseline model.

These visualization results highlight the superior robustness and discriminative capability of our proposed network in real-world foggy environments, which is especially beneficial for navigation assistance systems designed for visually impaired individuals. By improving the detection of difficult and ambiguous obstacles, our model provides a safer and more reliable perception foundation for assistive technologies in adverse weather conditions.

## 6. Discussion

Although our method achieved promising results and was effectively validated on both the RTTS and Foggy Cityscape datasets—demonstrating strong generalization capability—several issues remain to be addressed in future work:

1. Evaluation under varying fog densities:

Our current study did not include a systematic validation of the model’s robustness across datasets with different fog concentrations. In future work, we plan to evaluate the proposed method under controlled or annotated varying fog densities to more rigorously assess its stability and robustness.

2. Adaptation to other adverse weather conditions:

We intend to extend the applicability of our model to other challenging environments, such as heavy rain, heavy snow, and nighttime conditions, in order to further enhance the model’s adaptability and comprehensive robustness.

3. Model complexity and large-scale deployment:

Our goal is to conduct large-scale real-world validation across diverse navigation environments. To facilitate efficient deployment on mobile and wearable assistive devices, we will focus on optimizing the network by reducing its complexity and energy consumption, thereby enabling practical application in resource-constrained scenarios.

Through these improvements, we expect YOLO-Extreme to deliver even greater practical value, providing visually impaired individuals with safer, more reliable navigation assistance in a wide range of complex environments.

## 7. Conclusions

In this work, we proposed YOLO-Extreme, a novel and efficient object detection framework specifically designed to address the challenges of visually impaired navigation under foggy and complex urban environments. By introducing three core modules—the Dual-Branch Bottleneck Block (DBB), the Multi-Dimensional Collaborative Attention Module (MCAM), and the Channel-Selective Fusion Block (CSFB)—our approach effectively mitigates the issues of blurred object boundaries, low-contrast obstacles, and severe background noise.

Comprehensive experiments on both the RTTS foggy dataset and the Foggy Cityscape dataset demonstrate that YOLO-Extreme achieves state-of-the-art detection accuracy and real-time performance, consistently outperforming traditional dehaze-and-detect pipelines as well as classical object detectors. In particular, cross-dataset evaluation on Foggy Cityscape further validates the generalization capability of our proposed method to different foggy urban scenarios. Detailed ablation studies confirm the complementary benefits of the proposed modules for robust and precise detection in challenging weather conditions. These results underscore the practical value of YOLO-Extreme for providing safer and more reliable navigation assistance to visually impaired individuals in adverse real-world environments.

## Figures and Tables

**Table 1:** Ablation results mAP for different module combinations on the RTTS dataset.

Models	DBB	MCAM	CSFB	Person	Bus	Car	Motor	Bicycle	All
YOLOv12-n				0.507	0.445	0.581	0.446	0.434	0.482
	✓			0.520	0.447	0.596	0.471	0.441	0.495
		✓		0.526	0.449	0.597	0.453	0.436	0.492
			✓	0.523	0.467	0.603	0.459	0.433	0.497
	✓	✓		0.525	0.449	0.598	0.459	0.441	0.496
	✓		✓	0.524	0.452	0.600	0.463	0.442	0.498
		✓	✓	0.527	0.454	0.602	0.465	0.445	0.499
	✓	✓	✓	0.530	0.457	0.605	0.468	0.447	0.501

**Table 2:** Ablation results of FLOPs and parameters for different module combinations.

Models	DBB	MCAM	CSFB	Flops/G	Parameters/M
YOLOv12-n				5.8	2.51
	√			6.3	2.55
		√		6.3	2.56
			√	9.1	3.44
	√	√		6.4	2.56
	√		√	9.1	3.45
		√	√	9.2	3.45
	√	√	√	9.1	3.45

**Table 3:** Comparison with state-of-the-art methods on the RTTS dataset. All results are mAP@0.5:0.95 for each category and overall.

Method	Person	Bicycle	Car	Motor	Bus	All
Yolov8 [46]	0.623	0.387	0.465	0.273	0.161	0.381
Yolov8-C [46]	0.619	0.364	0.157	0.241	0.155	0.367
AOD-YOLOv8 [47]	0.598	0.358	0.407	0.233	0.130	0.345
MSBDN-YOLOv8 [48]	0.589	0.374	0.393	0.209	0.120	0.337
Griddehaze-YOLOv8 [8]	0.612	0.386	0.453	0.258	0.146	0.371
DCP-YOLOv8 [49]	0.621	0.393	0.417	0.237	0.139	0.361
IA-Yolo [37]	0.671	0.353	0.414	0.211	0.136	0.357
DSNet [50]	0.566	0.345	0.402	0.198	0.124	0.327
MS-DAYOLOv8 [51]	0.637	0.391	0.479	0.281	0.157	0.389
D-YOLO [12]	0.658	0.402	0.538	0.308	0.242	0.430
YOLOv12-n	0.507	0.434	0.581	0.446	0.445	0.482
YOLOv13-n [52]	0.511	0.429	0.576	0.437	0.437	0.478
Ours	0.530	0.447	0.605	0.468	0.457	0.501

**Table 4:** Efficiency comparison of different methods.

Method	Speed (s/image)	FPS	mAP
Yolov8	0.025	40.0	0.381
AOD-YOLOv8	0.135	7.4	0.345
MSBDN-YOLOv8	0.104	9.6	0.337
GridDehaze-YOLOv8	0.071	14.1	0.371
DS-Net	0.049	20.4	0.327
IA-YOLO	0.035	28.6	0.357
D-YOLO	0.033	30.3	0.430
YOLOv12-n	0.0027	365	0.482
YOLOv13-n	0.003	333	0.478
Ours	0.0043	232	0.501

**Table 5:** Comparison with state-of-the-art methods on the Foggy Cityscapes dataset [45].

Method	Person	Bicycle	Car	Motor	Bus	All
Yolov8	0.257	0.159	0.366	0.042	0.172	0.199
Yolov8-C	0.261	0.153	0.359	0.037	0.158	0.194
IA-YOLO	0.267	0.174	0.373	0.043	0.192	0.210
DSNet	0.251	0.143	0.369	0.045	0.167	0.195
MS-DAYolo	0.274	0.212	0.353	0.034	0.179	0.210
AOD-YOLOv8	0.236	0.144	0.332	0.031	0.129	0.174
MSBDN-YOLOv8	0.235	0.127	0.341	0.053	0.130	0.177
Griddehaze-YOLOv8	0.221	0.137	0.323	0.030	0.133	0.169
DCP-YOLOv8	0.243	0.141	0.339	0.032	0.161	0.183
YOLOv12-n	0.446	0.155	0.238	0.141	0.353	0.222
Ours	0.457	0.163	0.245	0.155	0.371	0.239

## Data Availability

The datasets used in this study are publicly available. The RTTS dataset can be accessed at https://www.kaggle.com/datasets/tuncnguyn/rtts-dataset (accessed on 17 June 2025), and the Foggy Cityscapes dataset is available at https://www.cityscapes-dataset.com/ (accessed on 17 June 2025). All data used in the analysis are contained within the article.
